# MIB1 upregulates IQGAP1 and promotes pancreatic cancer progression by inducing ST7 degradation

**DOI:** 10.1002/1878-0261.12955

**Published:** 2021-05-01

**Authors:** Bin Zhang, Xiang Cheng, Sudong Zhan, Xin Jin, Tao Liu

**Affiliations:** ^1^ Department of Digestive Surgical Oncology Union Hospital Tongji Medical College Huazhong University of Science and Technology Wuhan China; ^2^ Cancer Center Union Hospital Tongji Medical College Huazhong University of Science and Technology Wuhan China; ^3^ Department of Urology The Second Xiangya Hospital Central South University Changsha China

**Keywords:** IQGAP1, MIB1, pancreatic cancer, ST7

## Abstract

Despite recent progress in cancer treatment, the prognosis of patients with pancreatic cancer still remains poor. Pancreatic tumors are reported to display high molecular heterogeneity. Elucidating the molecular mechanisms underlying pancreatic cancer progression is essential for improving patient treatment and survival. The overexpression of E3 ubiquitin ligase mind bomb 1 (MIB1) was previously described in pancreatic cancer cells, where it enhanced tumor cell proliferation. However, the role of MIB1 in pancreatic cancer progression remains elusive. In the present study, we confirmed that MIB1 expression is elevated in pancreatic cancer tissues and that high levels of MIB associate with unfavorable prognosis. Overexpression of MIB1 enhanced proliferation and invasion of pancreatic cancer cells both *in vitro* and *in vivo*. We further investigated the molecular mechanisms downstream of MIB1 and observed for the first time that MIB1 targets suppressor of tumorigenicity 7 protein (ST7), previously described as suppressor of tumorigenicity, for proteasomal degradation. Furthermore, we found that ST7 suppressed tumor growth by downregulating IQ motif containing GTPase activating protein 1 (IQGAP1) in pancreatic tumor cells. Thus, these data show that MIB1 promotes pancreatic cancer progression by inducing ST7 degradation followed by downregulation of IQGAP1 in pancreatic cancer cells. In conclusion, our research shows that the MIB1/ST7/IQGAP1 axis is essential for pancreatic cancer progression, and MIB1 inhibition may serve as a novel therapeutic strategy in patients with pancreatic cancer.

## Graphical abstract

In this study, we show that MIB1 overexpression drives pancreatic cancer progression by targeting ST7 for proteasomal degradation. Furthermore, we identified ST7 as a critical factor suppressing tumor development and progression by negatively regulating IQGAP1 expression in pancreatic cancer. Our research shows that the MIB1/ST7/IQGAP1 axis is essential for pancreatic cancer progression, and MIB1 inhibition may serve as a novel therapeutic strategy in patients with pancreatic cancer.

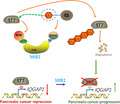

**Abbreviations**
DMEM: Dulbecco's modified Eagle’s medium
GTEx: Genotype‐Tissue Expression
IHC: immunohistochemistry
IP: immuno‐precipitated
IQGAP1: IQ motif containing GTPase activating protein 1
MAPK: mitogen‐activated protein kinase
MIB1: mind bomb 1
PLA: proximity ligation assay
qRT‐PCR: quantitative real‐time PCR
ST7: suppressor of tumorigenicity 7 protein
TCGA: The Cancer Genome Atlas
WCL: whole‐cell lysates
WRN: Werner syndrome

## 1 Introduction

Pancreatic tumors are extremely heterogeneous, characterized by fast growth, high metastatic potential, resistance to chemotherapy, and high mortality rate [1]. Genetic heterogeneity within tumors strongly affects numerous oncogenic signaling pathways, driving cancer evolution and resistance to treatment [2]. *KRAS*, *TP53*, *CDKN2A*, and *SMAD4* mutations have been linked to pancreatic cancer progression [3]. Despite recent progress in cancer therapeutics, the prognosis of pancreatic cancer patients remains extremely poor [4]. Therefore, elucidating the role of gene mutations and number alterations in the pathogenesis of pancreatic cancer is pivotal for improving the survival outcomes of patients with pancreatic cancer.

Mind bomb 1 (MIB1) is an E3 ubiquitin ligase involved in various cellular processes [5]. The N‐terminal of MIB1 contains two substrate recognition domains, whereas the C‐terminal of MIB1 mediates the degradation of substrates containing multiple RING domains [5]. MIB1 has been shown to ubiquitinate Delta proteins, which are Notch ligands [5]. In addition to regulating Notch signaling, MIB1 ubiquitinates other critical signaling components, such as degrading polo‐like kinase 4 to regulate centriole biogenesis [6]. Although mounting evidence suggests that MIB1 is overexpressed in pancreatic tumors [7], the role of MIB1 in the progression of pancreatic cancer remains unclear.

*KRAS* mutations are found in 90% of pancreatic cancer patients [4]. Aberrant mitogen‐activated protein kinase (MAPK) signaling activation is a key driver of cell proliferation and metastasis of pancreatic tumors [8]. IQ motif containing GTPase activating protein 1 (IQGAP1) has been identified as a scaffold protein regulating numerous MAPK cascades [9]. We have recently shown that FBP1 binds the WW domain of IQGAP1, inhibiting ERK phosphorylation in pancreatic cancer cells [10]. Nevertheless, the mechanisms regulating IQGAP in pancreatic cancer are understudied. Herein, we show that suppressor of tumorigenicity 7 protein (ST7) is a bona fide substrate of MIB1. MIB1 promotes ST7 degradation via the ubiquitin–proteasome system. We also report that in pancreatic cancer, ST7 suppresses MIB1 expression. Finally, we show that the MIB1/ST7 axis modulates IQGAP1 expression in pancreatic cancer. The results presented herein suggest that MIB1 is a promising candidate target for pancreatic cancer therapy.

## 2 Materials and methods

### 2.1 Cell lines and cell transfection

All pancreatic cancer cell lines (HPDE6‐C7, PANC‐1, BxPC‐3, AsPC‐1, SW1990, MIA PaCa‐2) were obtained from the Chinese Academy of Science Cell Bank, as previously reported [11]. Cells were maintained in Dulbecco’s modified Eagle’s medium (DMEM; Invitrogen, Carlsbad, CA, USA) containing 10% FBS (HyClone, Logan, UT, USA) in 5% CO_2_ and 37 °C humified atmosphere. Transfections were performed using Lipofectamine 2000 (Thermo Fisher Scientific, Waltham, MA, USA). Control shRNA and shMIB1#1, shMIB1#2, shST7#1, and shST7#2 (Sigma‐Aldrich, Shanghai, China) were employed to produce different lentiviral particles in 293T cells. After a 24‐h incubation, the cell culture medium was refreshed (DMEM containing 10% FBS and 1 mm sodium pyruvate). Subsequently, the virus‐containing cell culture medium was harvested after 48 h and used for pancreatic cancer cell transduction after the addition of 12 μg·mL^−1^ polybrene. Puromycin selection (10 μg·mL^−1^; 24 h) was performed to eliminate noninfected cells. The sequences of the shRNAs used are provided in Table S2.

### 2.2 Plasmids and reagents

Flag‐MIB1, MIB1 N‐terminal, and MIB1 C‐terminal plasmids were cloned into the CMV‐MCS‐3xFlag‐SV40‐neomycin vector by GENECHEM (Shanghai, China). The Flag‐MIB1ΔRING mutant was generated using a KOD‐Plus‐Mutagenesis Kit (Cat #SMK‐101B; TOYOBO, Osaka, Japan). The following primary antibodies were used in our study: MIB1 (4400; Cell Signaling Technology, Shanghai, China; 1 : 1000 dilution), GAPDH (ab8245; Abcam, Shanghai, China; 1 : 5000 dilution), ST7 (ab122459; Abcam; 1 : 200 dilution), and IQGAP1 (20648; Cell Signaling Technology; 1 : 2000 dilution). The proteasome inhibitor MG‐132 (Cat. No. S2619) was purchased from Selleckchem, Shanghai, China.

### 2.3 Co‐immunoprecipitation and immunoblotting

The study was conducted in accordance with the principles of the Declaration of Helsinki principles. It was approved by the Animal Use and Care Committees at Tongji Medical College, Huazhong University of Science and Technology. Ethical approval for the use of human tissues (12 pairs of matched pancreatic cancer and adjacent noncancerous tissues) was obtained by the local ethics committee (Tongji Medical College, China). The study methodologies conformed to the standards set by the Declaration of Helsinki. Written informed consent was acquired from all patients before surgery. For co‐immunoprecipitation, cells were harvested and incubated in 1 mL of RIPA buffer for 20 min on ice. Cell lysates were centrifuged at 15  000 ***g*** for 15 min at 4 °C. The supernatant after centrifugation was collected and incubated with Pierce Protein G Agarose (Thermo Fisher Scientific) and primary antibody or IgG at 4 °C overnight. The beads were washed five times with RIPA buffer, resuspended with loading buffer, and boiled at 100 °C for 5 min. The supernatant was subjected to immunoblotting. Pancreatic cancer whole‐cell lysates (WCL) were obtained in RIPA buffer, freshly supplemented with 1 mm phenylmethanesulfonyl fluoride. Protein concentration was assessed by the BCA method. Equal amounts of protein were resolved by SDS/PAGE and transferred onto PVDF membranes. Subsequently, membranes were incubated with primary antibodies for more than 8 h at 4 °C. Next, membranes were probed with the appropriate secondary antibody for 1 h at room temperature. Signal intensities were measured using the Chemiluminescent Western Blot Detection Kit (cat. no. 32209; Thermo Fisher Scientific).

### 2.4 Proximity ligation assay (PLA)

The BxPc‐3 cells were fixed by the blocking solution following the manufacture’s protocol (Duolink in situ fluorescence; Sigma). Then, the primary antibodies MIB1 (sc‐393551; Santa Cruz Biotechnology, Beijing, China; 1 : 200 dilution) and ST7 (ab122459; Abcam; 1 : 100 dilution), or IgG (Rabbit) (3900; Cell Signaling Technology; 1 : 5000 dilution) and IgG (Mouse) (53484; Cell Signaling Technology; 1 : 5000 dilution) were applied to incubated with the cells for 2 h at 37 °C. Then, cells were washed with 1× wash buffer and incubated with PLA probe for 1 h at 37 °C. The ligation–ligase was added to cells at 37 °C. Thirty minutes later, cells were incubated with amplification–polymerase solution for 100 min. The Duolink In Situ Mounting Medium with DAPI was added to cells to take photos under confocal microscope.

### 2.5 Quantitative real‐time PCR (qRT‐PCR)

Total RNA from pancreatic cancer cells was extracted using TRIzol (Thermo Fisher Scientific). cDNA was generated by reverse transcription (PrimeScript™ RT reagent Kit) as reported previously [12]. qRT‐PCR analyses were carried out using TB Green™ Fast qPCR Mix. Relative expression levels of genes of interest were determined using the 2^‐ΔΔCq^ method after normalization to *GAPDH* mRNA levels. The sequences of the qRT‐PCR primers for *MIB1, ST7, IQGAP1,* and *GAPDH* are provided in Table S1.

### 2.6 *In vitro* cell growth assay

Pancreatic cancer cells (1 × 10^4^) were seeded in 96‐well plates, and MTS solution was added according to the manufacturer’s instructions (cat. no. ab197010; Abcam). Absorbance at 490 nm was measured to evaluate *in vitro* cell growth. For colony formation assay, the cells were seeded in 6‐well plates (500 cells/well) and incubated in complete growth medium containing 10% FBS at 37 °C. Fourteen days later, the cells were fixed in methanol for 30 min and stained with 1% Crystal Violet Staining Solution for 30 min and then washed with PBS three times. Finally, the number of colonies was calculated.

### 2.7 *In vitro* invasion assay

*In vitro* cell invasion assays were performed using Bio‐Coat Matrigel invasion chambers (**BD Biosciences**, Beijing, China) according to the manufacturer’s instructions. Briefly, cells were grown in the chamber inserts for 24 h and then were fixed in methanol for 15 min and stained with crystal violet (1 mg·mL^‐1^) for 20 min. Invading cells were counted in at least three fields per group.

### 2.8 *In vivo* tumor growth assay

Ethical approval was obtained by the Ethics Committee of Tongji Medical College, Huazhong University of Science and Technology for all animal procedures. CAnN. Cg‐Foxn1nu/Crl for BALB/c nude mice (4–5 weeks old, 18–20 g) was obtained from Vitalriver (Beijing, China). All mice were housed in standard conditions with a 12‐h light/dark and access to food and water *ad libitum*. BxPC‐3 cells were transduced with different lentiviral particles. After puromycin selection for 48 h, cells (1 × 10^7^ per mouse) were subcutaneously injected into the back of mice. The length and width of xenografts were measured using a Vernier caliper, and tumor volumes were calculated using the formula (L × W^2^)/2. At the study endpoint, mice were euthanized, and tumors were excised and weighted.

### 2.9 Tissue microarray and immunohistochemistry (IHC)

Tissue microarray (cat. no. XT14‐029; Outdo Biobank, Shanghai, China) and IHC were performed to assess the levels of MIB1 in pancreatic ductal adenocarcinoma (PDAC), as well as the relationship between MIB1 and ST7. For IHC, the following antibodies were used: MIB1 (4400; Cell Signaling Technology; 1 : 400 dilution) and ST7 (ab122459; Abcam; 1 : 200 dilution). The IHC score was calculated based on the staining intensity and the proportion of positive tumor cells. The staining intensity was scored as follows: 1 = weak staining at 100× magnification and limited or no staining at 40× magnification; 2 = moderate staining at 40× magnification; 3 = strong staining at 40× magnification. Two experienced and blinded to the study pathologists independently determined the IHC scores.

### 2.10 Correlation analysis using GEPIA

RNA sequencing data from The Cancer Genome Atlas (TCGA) and the Genotype‐Tissue Expression (GTEx) databases were analyzed using Gene Expression Profiling Interactive Analysis (GEPIA, http://gepia.cancerpku.cn/index.html). GEPIA performs survival analyses based on gene expression levels and uses a log‐rank test for hypothesis evaluation. GEPIA also performs a pairwise gene correlation analysis for any given sets of TCGA and GTEx expression data using Pearson correlation statistics.

### 2.11 Statistical analysis

Data were expressed as means ± SD. Statistical significance was determined by one‐way or two‐way ANOVA using graphpad prism 5 software, San Diego, CA, USA. A statistical significance threshold of *P*‐values < 0.05 was used.

## 3 Results

### 3.1 MIB1 overexpression is correlated with poor prognosis in pancreatic cancer

**Fig. 1:**
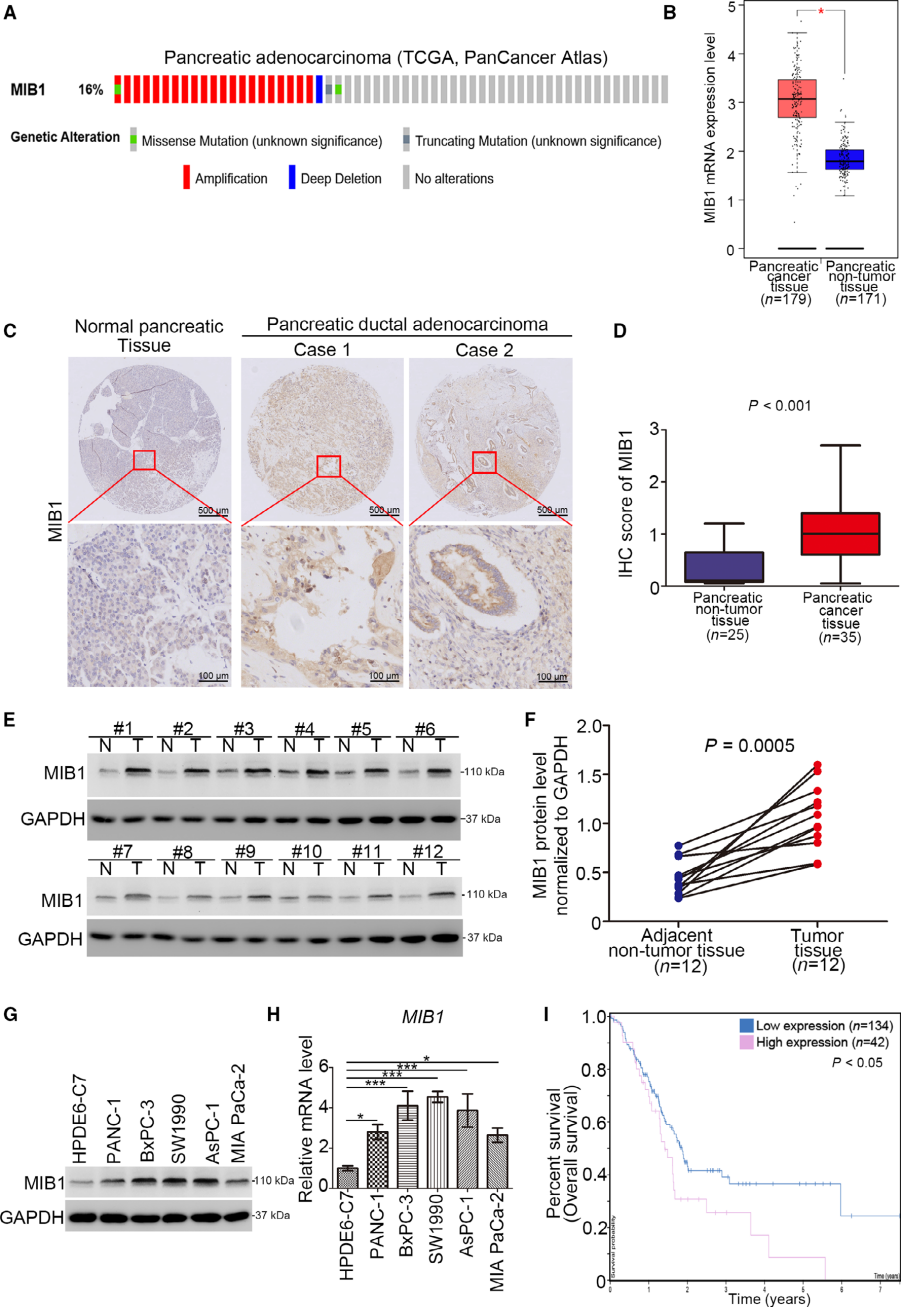
Aberrantly overexpressed MIB1 is associated with poor prognosis in pancreatic cancer. (A) The mRNA expression level of MIB1 in the TCGA dataset (http://www.cbioportal.org/). (B) Analysis of the mRNA expression level of MIB1 by using the GEPIA web tool (http://gepia.cancer‐pku.cn/). Wilcoxon signed‐rank test was used to determine the statistical significance. The error bar indicates SD. *, *P* < 0.01. (C) Representative IHC images stained with MIB1 in the tissue microarray. The scale bar as indicated in the figure. (D) The protein levels of MIB1 from the tissue microarray were determined by IHC analysis, Wilcoxon signed‐rank test was used to determine the statistical significance. *P* value as indicated in the figure. The error bar indicates SD. (E, F) The protein level of MIB1 from pancreatic cancer tissues (*n* = 12) and adjacent normal pancreatic tissues (*n* = 12) was detected by western blotting analysis, *P* = 0.0005. Wilcoxon signed‐rank test was used to determine the statistical significance. *P* value as indicated in the figure. (G, H) HPDE6‐C7, PANC‐1, BxPC‐3, SW1990, AsPC‐1, and MIA PaCa‐2 were harvested for western blotting analysis (G), RT‐qPCR analysis (H). For panel H, data presented as mean ± SD with three replicates (*n* = 3). Statistical significance was determined by one‐way ANOVA. *, *P* < 0.05; ***, *P* < 0.001. (I) The overall survival rate in high/low MIB1 group was analyzed by the human protein atlas (http://www.proteinatlas.org/), Log‐rank test was used to determine the statistical significance. *P* < 0.05.

To assess the association between MIB1 expression levels and pancreatic cancer clinicopathological characteristics, we evaluated *MIB1* mRNA levels in the TCGA database and found that *MIB1* was upregulated in 16% of pancreatic cancers (Fig. 1A). Consistently, we found that *MIB1* mRNA levels were significantly higher in pancreatic tumors (*n* = 179) than normal pancreatic tissues (*n* = 171) (Fig. 1B). We also investigated the protein levels of MIB1 in pancreatic cancer specimens using tissue microarrays (*n* = 25 normal pancreatic tissues, *n* = 35 PDAC) and IHC. MIB1 protein levels were elevated in pancreatic cancer tissues compared with normal pancreatic tissues (*P* < 0.001; Fig. 1C,D). Western blot analysis of specimens from our hospital [11] confirmed the higher MIB1 protein levels in pancreatic cancer (*n* = 12) than in adjacent nonmalignant tissues (*n* = 12) (Fig. 1E,F). Moreover, MIB1 expression levels were elevated in pancreatic cancer cell lines compared with nonmalignant pancreatic ductal epithelial cells (HDPE6‐C7; Fig. 1G,H). These results strongly indicate that MIB1 is upregulated in pancreatic cancer. Importantly, high MIB1 expression levels in pancreatic cancer patients were associated with shorter overall survival by using The Human Protein Atlas (Fig. 1I), suggesting that aberrant MIB1 expression may represent a poor prognosis biomarker in pancreatic cancer.

### 3.2 MIB1 promotes pancreatic cancer cell proliferation and invasion

**Fig. 2:**
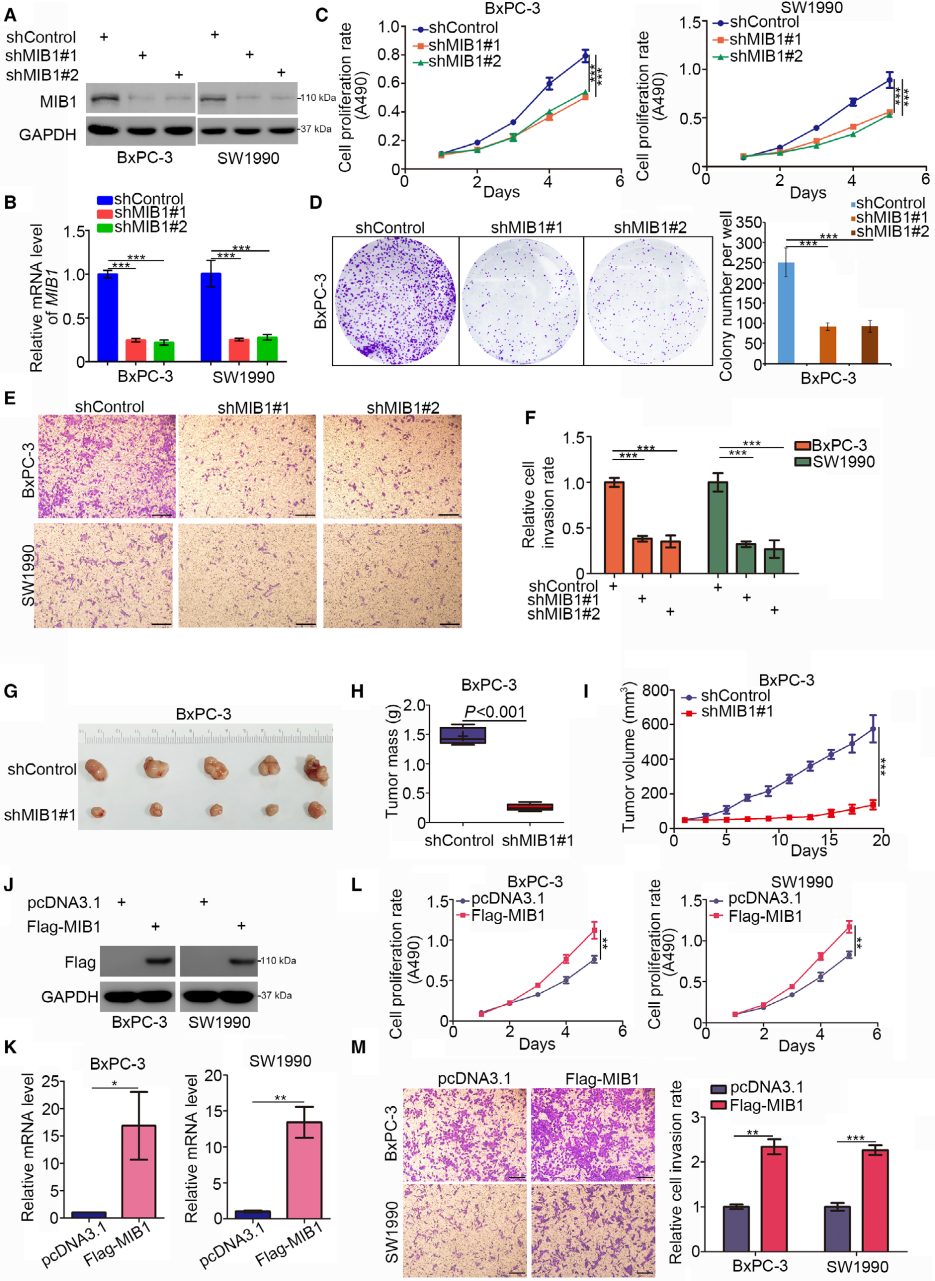
MIB1 is contributed to promoting pancreatic cancer cells progression. (A‐F), BxPC‐3 and SW1990 cells were infected with indicated shRNAs. 72 h post‐infection, cells were harvested for western blotting analysis (A), RT‐qPCR analysis (B), MTS assay (C), colony formation assay (D), and transwell assay (E). For panel B‐E, data presented as mean ± SD with three replicates. Statistical significance was determined by one‐way ANOVA. ***, *P* < 0.001. For panel E, the size of the scale bar on microscopy images was 100 μm. (G‐I) BxPC‐3 cells were infected with indicated shRNAs. After 72 h puromycin selection, cells were harvested and subcutaneously injected into nude mice for xenografts assay. The image of tumor was shown in panel G. The tumor mass was demonstrated in panel H. The tumor growth curve was indicated in panel I. Data presented as mean ± SD with five replicates. Student's t test was used to determine the statistical significance. ***, *P* < 0.001. (J‐M) BxPC‐3 and SW1990 cells were transfected indicated constructs. After 48 h, cells were harvested for western blotting analysis (J), RT‐qPCR analysis (K), MTS assay (L), and transwell assay (M). For panel K‐M, data presented as mean ± SD with three replicates. Student's *t* test was used to determine the statistical significance. *, *P* < 0.05; **, *P* < 0.01; ***, *P* < 0.001. For panel M, the size of the scale bar on microscopy images was 100 μm.

Next, we assessed MIB1’s role in pancreatic cancer cell growth. To this end, we silenced MIB1 expression in BxPC‐3 and SW1990 cells using two different shRNAs (Fig. 2A,B), followed by MTS assay and colony formation analysis. MIB1 silencing significantly inhibited cancer cell proliferation (Fig. 2C,D). Additionally, transwell assays revealed that MIB1 knockdown markedly impaired the invasion ability of cancer cells (Fig. 2E,F). To determine the impact of MIB1 knockdown on pancreatic cell growth *in vivo*, we subcutaneously injected BxPC‐3 cells (expressing shControl or shMIB1#1) in nude mice and assessed tumor growth. We found that MIB1 knockdown profoundly reduced tumor growth (Fig. 2G‐I). Conversely, ectopic overexpression of MIB1 (Fig. 2J,K) enhanced pancreatic cancer cell proliferation and invasion, both in BxPC‐3 and in SW1990 cells (Fig. 2L,M). These findings highlight MIB1’s key role in pancreatic cancer progression.

### 3.3 MIB1 interacts with ST7 in pancreatic cancer cells

**Fig. 3:**
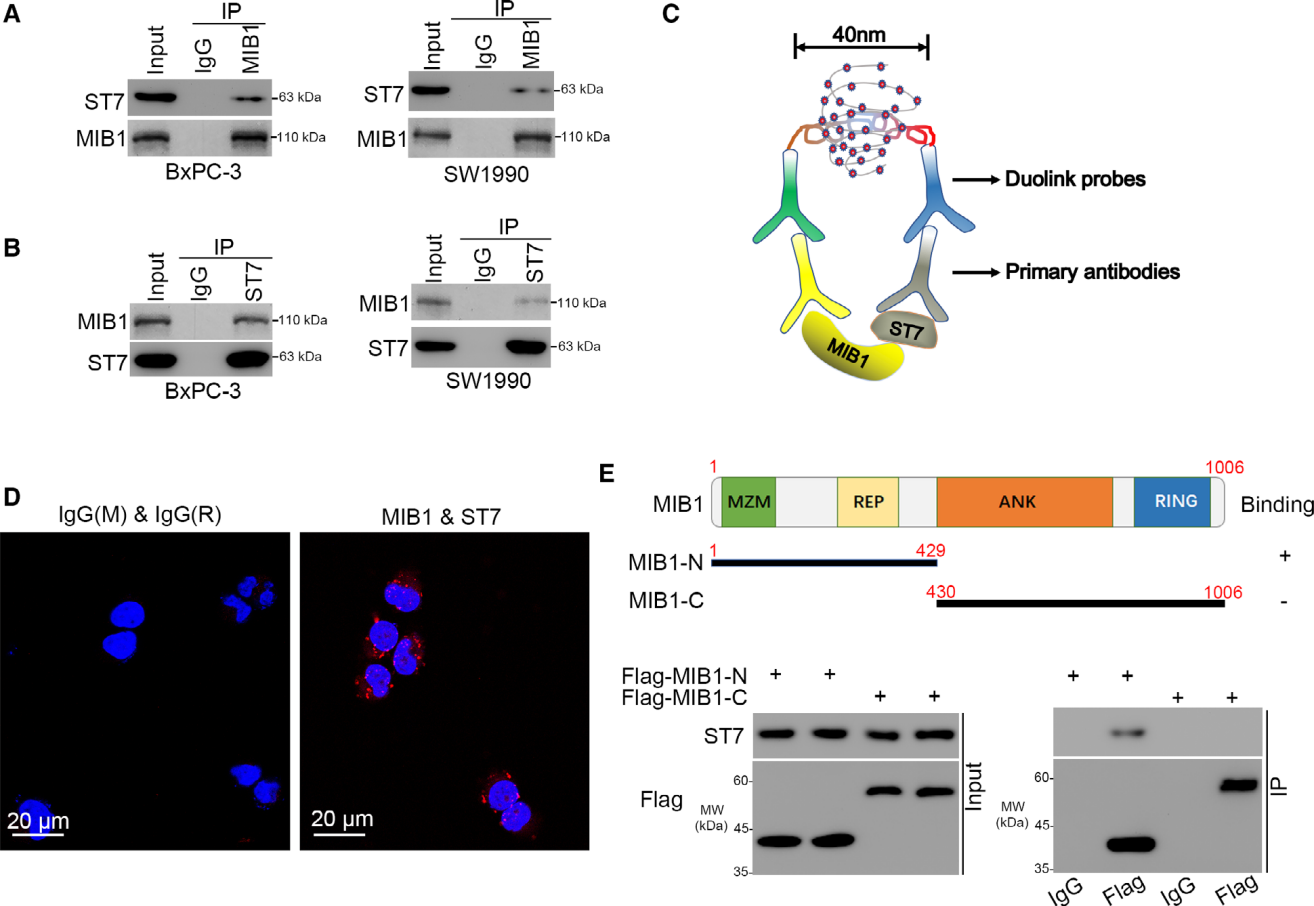
MIB1 interacts with ST7 in pancreatic cancer cells. (A, B) western blotting analysis of the WCL of BxPC‐3 and SW1990 cells. (C, D), the PLA by using the indicated antibodies (panel C) to verify the interaction between MIB1 and ST7 in BxPC‐3 cells. The size of the scale bar on microscopy images was 20 μm. (E) A schematic diagram depicting the domain of MIB1. (F) Flag‐MIB1‐C and Flag‐MIB1‐N were translated *in vitro*; the immunoprecipitation was employed to examine the region of MIB1 binding with ST7.

To elucidate the mechanisms underlying MIB1’s pro‐tumorigenic effects in pancreatic cancer, we performed immunoprecipitation and mass spectrometry to identify binding partners of MIB1 (Fig. S1A,B, and Table S3). Since MIB1 is an E3 ligase, we hypothesized that it promotes cancer development and progression by targeting tumor suppressor proteins for degradation. ST7 is known to suppress the growth of multiple solid tumor types, including prostate [13], gastric [14], and colorectal cancer [15]. ST7 was one of the potential binding partners of MIB1 (Fig. S1C). Co‐immunoprecipitation results confirmed the interaction between MIB1 and ST7 in BxPC‐3 and Sw1990 cells (Fig. 3A,B). Moreover, we performed a proximity ligation assay (PLA) to confirmed the interaction between MIB1 and ST7 in BxPC‐3 cells (Fig. 3C,D). Furthermore, to identify which MIB1 domain is responsible for its interaction with ST7, we constructed MIB1 N‐terminal (amino acids 1–429) and MIB1 C‐terminal (amino acids 430–1006) plasmids (Fig. 3E). We found that the N‐terminal domain of MIB1 was responsible for MIB1‐ST7 interaction (Fig. 3F).

### 3.4 ST7 is a bona fide substrate of MIB1 in pancreatic cancer

**Fig. 4:**
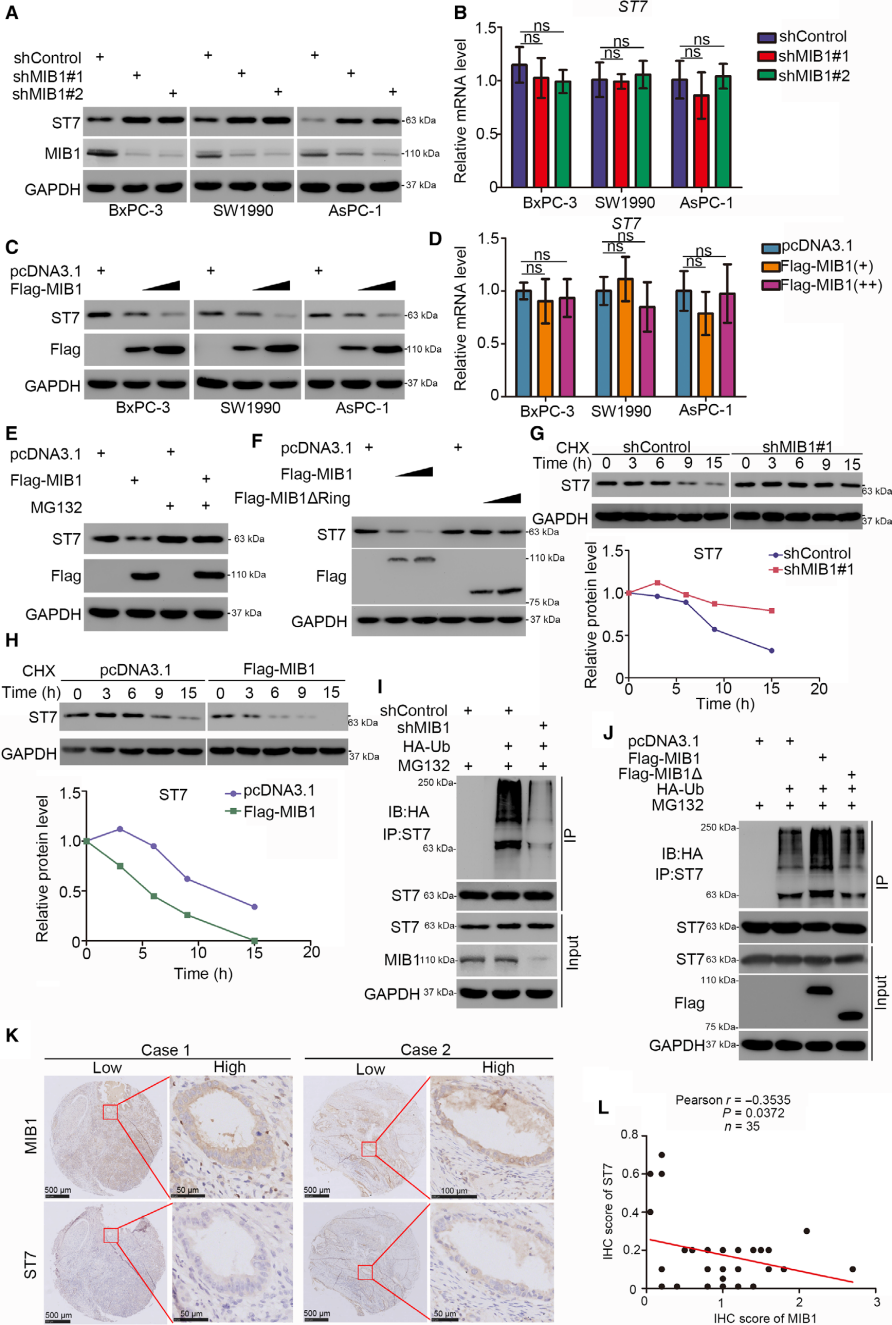
ST7 is a bona fide substrate of MIB1 in pancreatic cancer. (A and B) The pancreatic cancer cell lines (BxPC‐3, SW1990, AsPC‐1) were infected with indicated shRNAs. 72 h post‐infection, cells were harvested for western blotting analysis (A) and RT‐qPCR analysis (B). Data presented as mean ± SD with three replicates. Statistical significance was determined by one‐way ANOVA. ns, not significant. (C and D) The pancreatic cancer cell lines (BxPC‐3, SW1990, AsPC‐1) were transfected with indicated constructs. After 48 h, cells were harvested for western blotting analysis (C) and RT‐qPCR analysis (D). Data presented as mean ± SD with three replicates. ns, not significant. (E) BxPC‐3 cells were transfected with indicated constructs. After 48 h, the WCL of BxPC‐3 was subjected to western blotting analysis. Cells were treated with or without 20 µm of MG132 for 8 h before harvested. (F) BxPC‐3 cells were transfected with indicated constructs. After 48 h, the WCL of BxPC‐3 was subjected to western blotting analysis. (G) BxPC‐3 cells were infected with indicated shRNAs. After 72 h, cells were treated with cycloheximide (CHX) and cells were collected for western blotting analysis at different time points. (H) BxPC‐3 cells were transfected with indicated plasmids. After 48 h, cells were treated with cycloheximide (CHX) and cells were collected for western blotting analysis at different time points. The imagej software Laboratory for Optical and Computational Instrumentation (LOCI) of the University of Wisconsin‐Madison, Madison, WI, USA was used to quantified the protein level of ST7 and GAPDH. The relative protein level of ST7 to GAPDH was shown. (I) BxPC‐3 cells were infected with indicated plasmids. Seventy‐two hours postinfection, cells were collected for western blotting analysis after treated with MG132 for 8 h. The imagej software was used to quantified the protein level of ST7 and GAPDH. The relative protein level of ST7 to GAPDH was shown. (J) BxPC‐3 cells were transfected with indicated plasmids. After 48 h, cells were collected for western blotting analysis after treated with MG132 for 8 h. (K and L) The tissue microarray of pancreatic cancer was stained with MIB1 and ST7, respectively (*n* = 35). The typical IHC images stained with MIB1 and ST7 were shown in panel K. The size of the scale bar on microscopy images as indicated in the figure. The correlation of these two proteins was shown in panel L. Pearson correlation was used to determine statistical significance; the *P* value was indicated in the figure.

Given that MIB1 is an E3 ligase, we assessed whether MIB1 targets ST7 for degradation. We found that MIB1 knockdown increased the protein but not the mRNA level of ST7 in BxPC‐3, SW1990, and AsPC‐1 cells (Fig. 4A,B). Conversely, MIB1 overexpression decreased ST7 protein levels in pancreatic cancer cell lines, although ST7 mRNA levels remained unchanged (Fig. 4C,D). Interestingly, proteasome inhibition with MG132 in BxPC‐3 cells abrogated the ability of MIB1 overexpression to decrease ST7 levels (Fig. 4E). Since the E3 ligase activity of MIB1 requires the RING domain, we constructed a MIB1‐ΔRING mutant, which lacked the RING domain. Although the expression of wild‐type MIB1 decreased ST7 levels in BxPC‐3 cells, expression of the MIB1‐ΔRING mutant failed to do so (Fig. 4F). We also found that MIB1 repression prolonged the half‐life of ST7 protein (Fig. 4G) and reduced the polyubiquitination levels of ST7 in BxPC‐3 cells (Fig. 4I). Conversely, the protein half‐life of ST7 in MIB1 overexpressing cells was shorter than that in the control group (Fig. 4H). Moreover, forced expression of wild‐type MIB1 increased ST7’s polyubiquitination level, although expression of the MIB1ΔRING mutant did not alter the levels of ST7 polyubiquitination in BxPC‐3 cells. We also evaluated the protein levels of ST7 and MIB1 in a pancreatic cancer tissue microarray (*n* = 35 samples). We found a reverse association between MIB1 and ST7 protein levels in pancreatic cancer tissues (Pearson correlation *r* = −0.3535, *P* = 0.0372; Fig. 4K.). Together, these data indicate that MIB1 promotes ST7 polyubiquitination and proteasomal degradation in pancreatic cancer.

### 3.5 ST7 plays a key role in MIB1‐induced pancreatic cancer cell growth

**Fig. 5:**
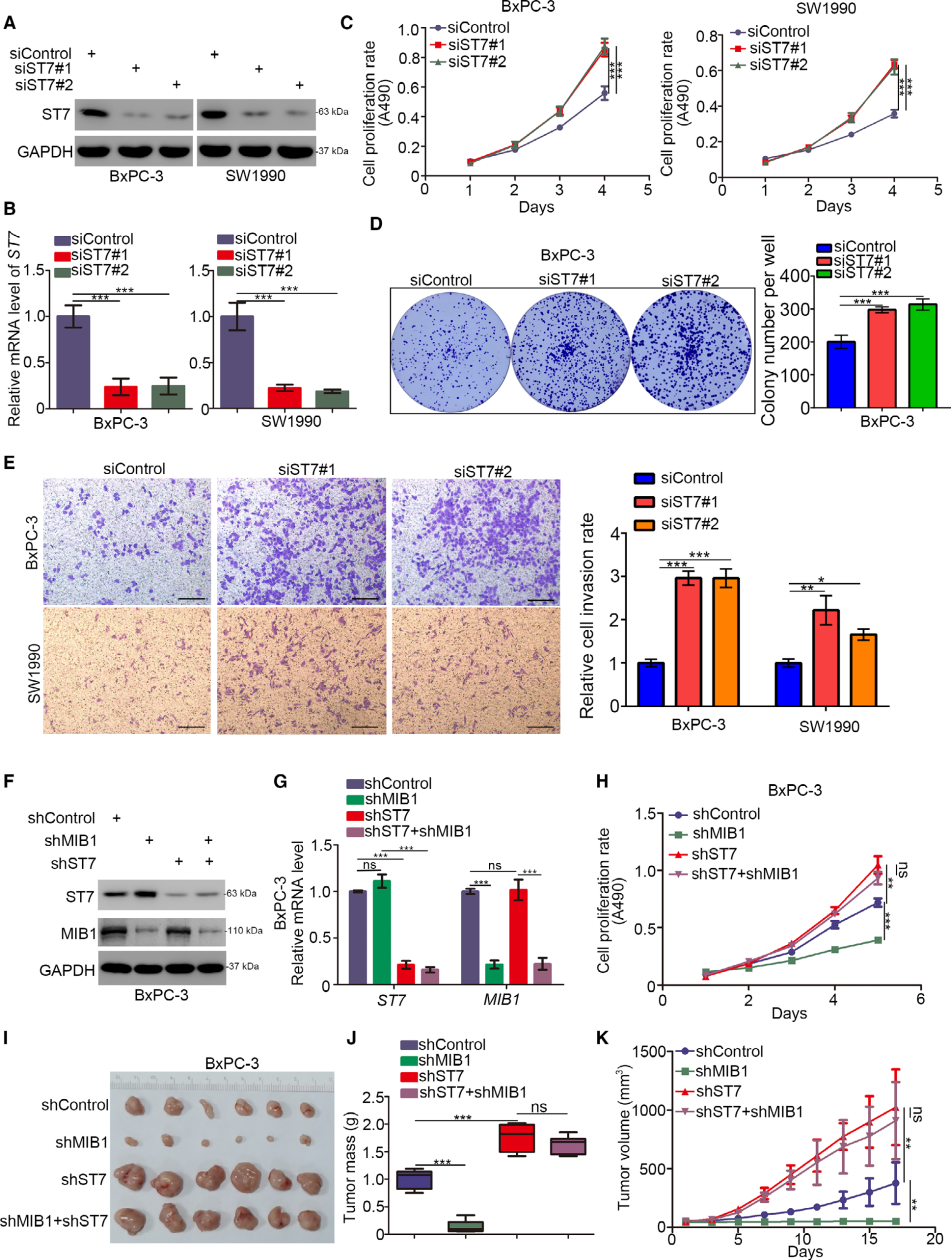
ST7 is the key mediator for MIB1‐induced pancreatic cancer cells progression. (A‐E) BxPC‐3 and SW1990 cells were infected with indicated shRNAs. Seventy‐two hours post‐infection, cells were harvested for western blotting analysis (A), RT‐qPCR analysis (B), MTS assay (C), colony formation assay (D), and transwell assay (E). For panel B‐E, data presented as mean ± SD with three replicates. Statistical significance was determined by one‐way ANOVA. *, *P* < 0.05; **, *P* < 0.01; ***, *P* < 0.001. For panel E, the size of the scale bar on microscopy images was 100 μm. (F and K), BxPC‐3 were infected with indicated shRNAs. Seventy‐two hours post‐infection and puromycin selection, cells were harvested for western blotting analysis (F), RT‐qPCR analysis (G), MTS assay (H), and xenograft assay (I‐K). For panel G and H, data presented as mean ± SD with three replicates (*n* = 3). Statistical significance was determined by one‐way ANOVA. ns, not significant; **, *P* < 0.01; ***, *P* < 0.001. The image of tumor was shown in panel I. The tumor mass was demonstrated in panel J. The tumor growth curve was indicated in panel K. Data presented as mean ± SD with five replicates (*n* = 5). Statistical significance was determined by one‐way ANOVA. ns, not significant; **P < 0.01;***P < 0.001.

Although ST7 has been reported to suppress tumor growth in multiple cancer entities, its role in pancreatic cancer remains unknown. MTS and colony formation assays revealed that ST7 silencing by two different siRNAs (Fig. 5A,B) markedly increased cell growth, both in BxPC‐3 and in SW1990 cells (Fig. 5C,D). Similarly, ST7 knockdown enhanced pancreatic cancer cell invasion *in vitro* (Fig. 5E). These findings suggest that ST7 acts as a tumor suppressor in pancreatic cancer. To assess the importance of ST7 in the tumor‐promoting effects of MIB1 in pancreatic cancer, we used BxPC‐3 cells expressing shControl, shMIB1, shST7, and shMIB1/shST7 (Fig. 5F,G). ST7 silencing increased tumor growth, and MIB1 silencing alone inhibited the proliferation of BxPC‐3 cells (Fig. 5H‐K). Notably, combined silencing of ST7 and MIB1 diminished the antiproliferative effects of MIB1 downregulation *in vitro* and *in vivo* (Fig. 5H‐K), indicating that MIB1 promotes pancreatic cancer progression by targeting ST7 for degradation.

### 3.6 The MIB1/ST7/IQGAP1 signaling axis promotes pancreatic cancer proliferation

**Fig. 6:**
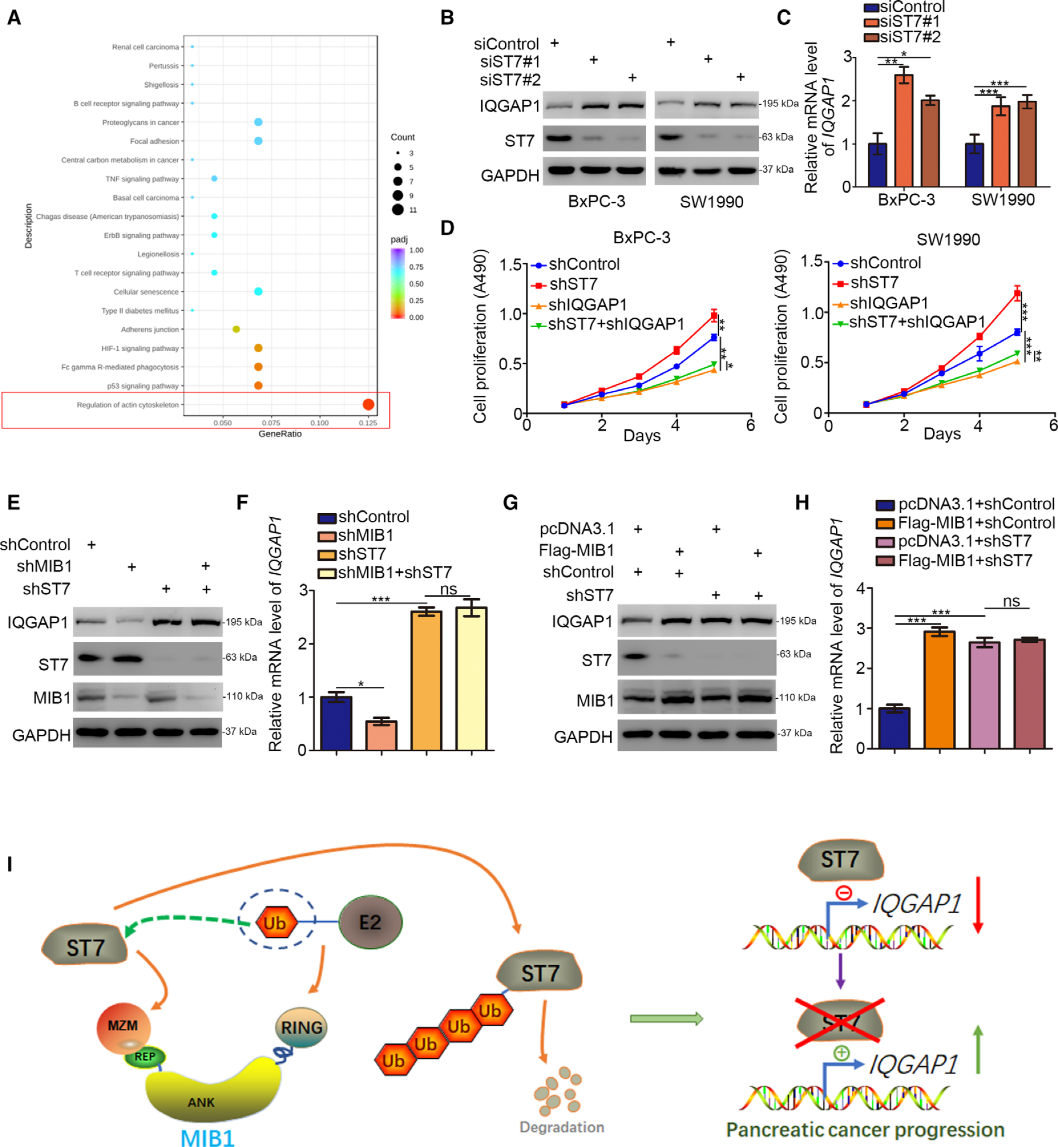
The MIB1/ST7/IQGAP1 signaling axis increases pancreatic cancer proliferation. (A) BxPC‐3 cells were transfected with indicated constructs for 48 h. Cells were subjected to RNA‐seq analysis and subsequent KEGG pathway enrichment. (B and C) BxPC‐3 and SW1990 cells were transfected with indicated constructs. After 48 h, cells were harvested for western blotting analysis (B) and RT‐qPCR analysis (C). Statistical significance was determined by one‐way ANOVA. Data presented as mean ± SD with three replicates (*n* = 3). *, *P* < 0.05; **, *P* < 0.01; ***, *P* < 0.001. (D) BxPC‐3 and SW1990 cells were infected with indicated shRNAs. Seventy‐two hours postinfection, cells were harvested for MTS assay. Statistical significance was determined by one‐way ANOVA on the fifth day. Data presented as mean ± SD with three replicates (*n* = 3). *, *P* < 0.05; **, *P* < 0.01; ***, *P* < 0.001. (E and F) BxPC‐3 cells were infected with indicated shRNAs. Seventy‐two hours postinfection, cells were harvested for western blotting analysis (E) and RT‐qPCR analysis (F). Data presented as mean ± SD with three replicates. ns, not significant; *, *P* < 0.05; ***, *P* < 0.001. (G and H) BxPC‐3 cells were infected with indicated shRNAs. After 48 h, cells were transfected with indicated plasmids for other 24 h. Cells were harvested for western blotting analysis (G) and RT‐qPCR analysis (H). Data presented as mean ± SD with three replicates. ns, not significant; ***, *P* < 0.001. (I) a hypothesis model depicting that abnormally upregulated MIB1 degrades ST7 to increase IQGAP1 expression and promotes pancreatic cancer progression.

To get further insight into the antitumor effects of ST7 in pancreatic cancer, we performed RNA‐seq analysis in BxPC‐3 cells with or without ST7 silencing (Fig. S2A,B, Table S4). KEGG pathway enrichment analysis of the differentially expressed genes demonstrated that ST7 knockdown upregulated numerous genes involved in the actin cytoskeleton pathway (Fig. 6A). Importantly, ST7 silencing significantly upregulated IQGAP1, which has been shown to promote pancreatic cancer proliferation [10]. We conducted western blotting and qRT‐PCR analysis and confirmed that ST7 knockdown increased IQGAP1 mRNA and protein levels; this finding was consistent in BxPC‐3 and SW1990 cells (Fig. 6B,C). Additionally, IQGAP1 silencing alone suppressed cell growth in BxPC‐3 and SW1990 cells (Fig. 6D). The ability of ST7 silencing to increase pancreatic cancer cell growth was attenuated by the combined silencing of IQGAP1 and ST7 (Fig. 6D). These results indicate that IQGAP1 is essential for the antitumor effects of ST7 in pancreatic cancer.

Next, we silenced ST7, IQGAP1, and ST7/IQGAP1 expression in BxPC‐3 cells. MIB1 knockdown decreased IQGAP1 expression levels; however, double knockdown of MIB1 and ST7 abolished the ability of MIB1 to regulate IQGAP1 expression (Fig. 6E,F). MIB1 overexpression upregulated IQGAP1 in BxPC‐3 cells, both at the protein and at mRNA levels (Fig. 6G,H). Consistently, ST7 knockdown abrogated the MIB1‐mediated IQGAP1 upregulation (Fig. 6G,H). Overall, these data indicate that MIB1 targets ST7 for degradation, thereby increasing IQGAP1 expression levels and promoting pancreatic cancer progression (Fig. 6I).

## 4 Discussion

ST7 mutations have been reported in breast, colon, esophagus, and gastric cancers [14, 16, 17]. Additionally, ST7 overexpression has been shown to suppress the *in vivo* tumorigenicity PC‐3 prostate cancer cells [16], suggesting that ST7 has tumor suppressor functions [18]. However, the precise mechanisms underlying the antitumor effects of ST7 are poorly understood. Previously studies have shown that ST7 suppressed tumor growth by modulating the expression of genes involved in cellular structure and architecture [18]. Consistently, our RNA‐seq analysis indicated that ST7 regulated the expression of actin cytoskeleton‐related genes in pancreatic cancer. In particular, we identified that IQGAP1, a well‐known cytoskeleton regulator [19, 20], was downregulated by ST7 in pancreatic cancer. Intriguingly, pancreatic cancers often exhibit *KRAS* mutation and constitutive activation [10]. In addition to regulating the cytoskeleton, IQGAP1 is a scaffold protein modulating MAPK pathway activation [21]. Thus, targeting the IQGAP1 axis may represent an attractive approach for the treatment of pancreatic cancer. Here, we found that the ST7/IQGAP1 axis plays an essential role in pancreatic cancer progression; nevertheless, the mechanism of how ST7 inhibits IQGAP1 requires further investigation.

MIB1 contains multiple RING domains and functions as an E3 ubiquitin ligase, regulating several cellular processes [5]. Notably, MIB1 binds the intracellular tails of Notch ligands, promoting their ubiquitination necessary for Notch signaling activation [5]. Given the important role of the Notch pathway in modulating tumor growth, cancer metastasis, and the tumor microenvironment, MIB1‐mediated ubiquitination of Notch ligands may affect all these tumor characteristics [22, 23, 24]. For instance, MIB1 has been shown to ubiquitinate JAG1 and activate Notch signaling in breast cancer [25]. Additionally, MIB1 has been implicated in the pathogenesis of spinal muscular atrophy by regulating cellular senescence and degrading motor neuron [26]) and Werner syndrome protein [27]. Recently, Fu *et al*. [7] reported that MIB1 enhances pancreatic cancer growth and regulates gemcitabine resistance by activating β‐catenin signaling . Nonetheless, the precise mechanisms of how MIB1 activates β‐catenin are still unknown. Our data demonstrated that MIB1 exerted oncogenic functions in pancreatic cancer. Importantly, we identified ST7 as a novel substrate of MIB1 in pancreatic cancer. Although the c‐Myc‐dependent CRL4^DCAF4^ E3 ligase has been shown to mediate ST7 degradation in colitis‐associated cancer [28], the post‐translational modifications of ST7 in pancreatic cancer remain unknown. In the present study, we identified that by inducing ST7 degradation, MIB1 upregulates IQGAP1 and enhances pancreatic cancer progression.

## 5 Conclusions

Our data suggest that MIB1 overexpression drives pancreatic cancer progression by targeting ST7 for degradation. Furthermore, we identified ST7 as a critical factor suppressing tumor development and progression in pancreatic cancer by negatively regulating IQGAP1 expression. Therefore, the MIB1/ST7/IQGAP1 signaling axis is an important mediator of pancreatic cancer progression, and inhibiting MIB1 may prolong the survival of patients with pancreatic tumors.

## Conflict of interest

The authors declare no conflict of interest.

## Author contributions

XJ contributed to investigation, methodology, project administration, and writing—original draft; BZ contributed to software, formal analysis, and methodology; XC contributed to conceptualization, supervision, and project administration; SZ contributed to software and methodology; TL contributed to funding acquisition, project administration, writing—original draft, and writing—review and editing.

## Consent for publication

All subjects have written informed consent.

### 1 Peer Review

The peer review history for this article is available at https://publons.com/publon/10.1002/1878-0261.12955.

## Supporting information


**Table S1**. Sequences of RT‐qPCR primers.
**Table S2**. Sequences of gene‐specific shRNAs.

**Table S3**. Mass spectrometry of MIB1.

**Table S4**. RNA‐seq of ST7.
**Fig. S1**. ST7 is the binding partner of MIB1.
**Fig. S2**. RNA‐seq analysis of BxPC‐3 cells.

## Data Availability

The datasets used and/or analyzed during the current study are available from the corresponding authors (Xin Jin, jinxinunion@hust.edu.cn) on reasonable request.
