# Supplementary material for: MIB1 upregulates IQGAP1 and promotes pancreatic cancer progression by inducing ST7 degradation
Source: Mol Oncol. 2021 May 1;15(11):3062–75. doi: 10.1002/1878-0261.12955 (PMC8564634; doi:10.1002/1878-0261.12955)
Supplement: Supplementary file 1 — Table S1. Sequences of RT‐qPCR primers. Table S2. Sequences of gene‐specific shRNAs. [file MOL2-15-3062-s001.docx]

**MIB1 upregulates IQGAP1 and promotes pancreatic cancer progression by inducing ST7 degradation**

Bin Zhang, Xiang Cheng, Sudong Zhan, Xin Jin, Tao Liu

**Table S1: Sequences of RT-qPCR primers**

| **Species** | **Gene** | **Forward (5’-3’)** | **Reverse (5’-3’)** |
| --- | --- | --- | --- |
| Human | *GAPDH* | CCAGAACATCATCCCTGCCT | CCTGCTTCACCACCTTCTTG |
| Human | *MIB1* | ATGTGCTGTGGAGGGAAAAG | GACACACAGGGCACATTGTC |
| Human | *ST7* | TCAATCCTCATGTGCCAAAA | TTCCCACGTACAATGCAAAA |
| Human | *IQGAP1* | GACAGGAGAGGCAAGCAAAC | TCAGCACTTTGGCAATGAAG |

**Table S2: Sequences of gene-specific shRNAs**

| shMIB1-1 | 5′- CCGGCCTCTGGGATAATGGTGCTAACTCGAGTTAGCACCATTATCCCAGAGGTTTTTG-3′ |
| --- | --- |
| shMIB1-2 | 5′- CCGGCAGAGGATAAAGATGGTGATACTCGAGTATCACCATCTTTATCCTCTGTTTTTG-3′ |
| shST7-1 | 5′- CCGGGTGGTTCTTCATCGTGCTATTCTCGAGAATAGCACGATGAAGAACCACTTTTTG-3′ |
| shST7-2 | 5′- CCGGGCTTCCCTTCTTTATTCTCTTCTCGAGAAGAGAATAAAGAAGGGAAGCTTTTTG-3′ |

**
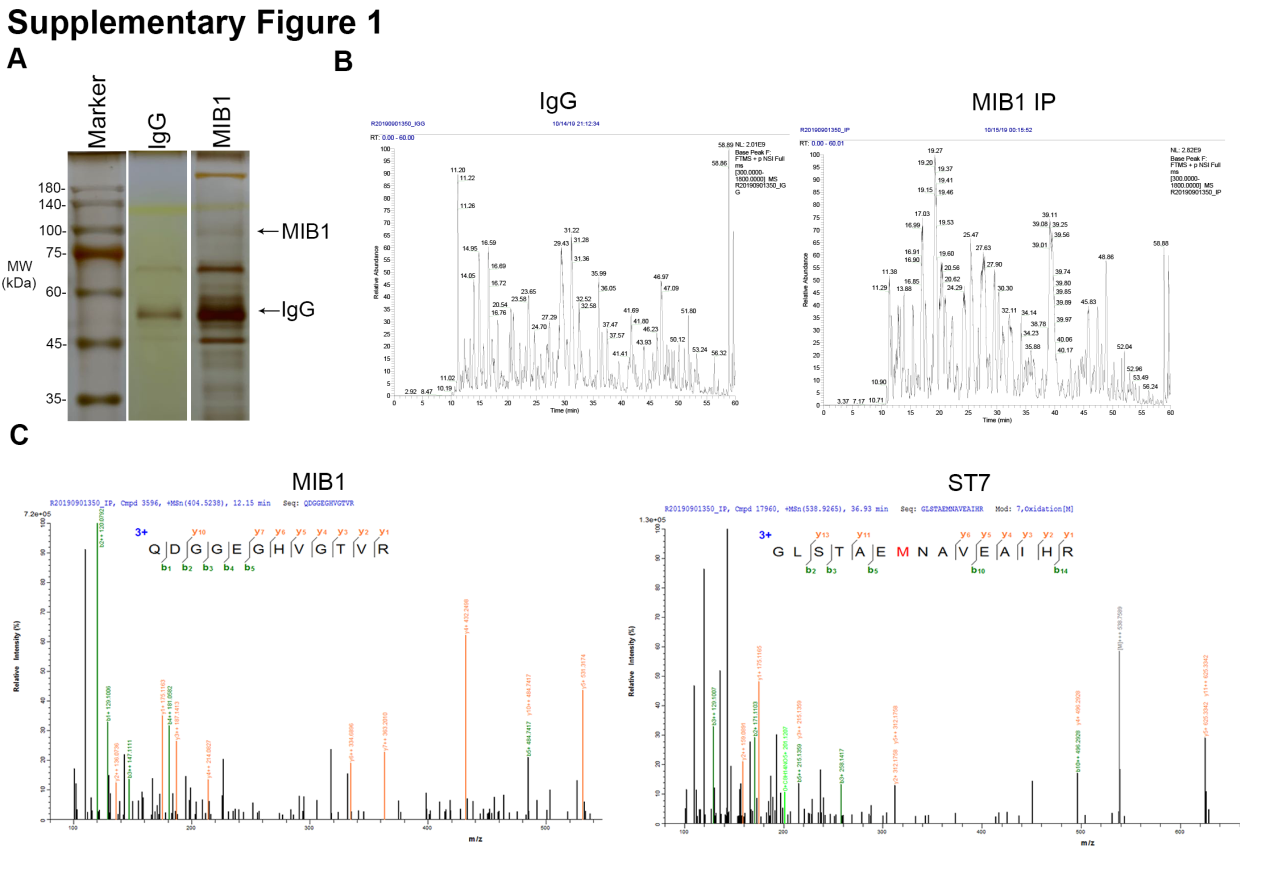
**

**Supplementary Figure 1. ST7 is the binding partner of MIB1.**

**A-C,** the WCL of BxPC-3 cells were subjected to silver staining (B) and mass spectrometry with IgG and MIB1 antibodies (B and C).

**
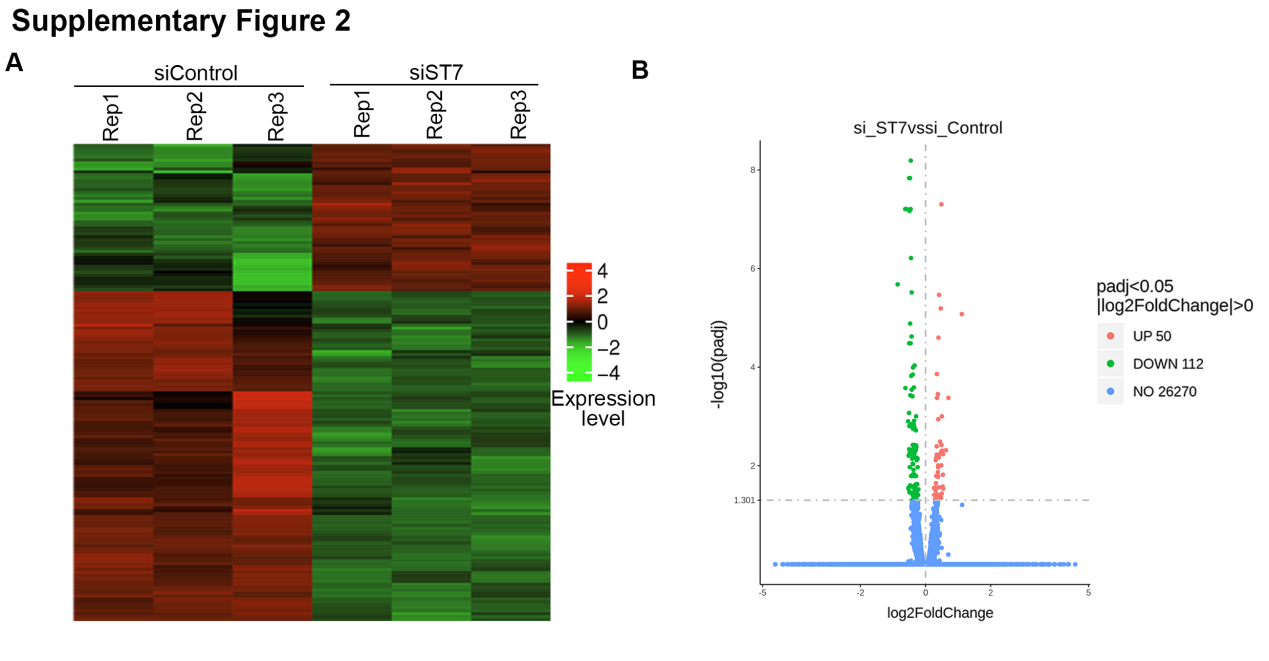
**

**Supplementary Figure 2. RNA-seq analysis of BxPC-3 cells**

**A and B，**the BxPC-3 cells were transfected with indicated constructs for 48h. Cells were subjected to RNA-seq analysis (A and B)
